# Effects of low-dose combined olanzapine and sertraline on negative and depressive symptoms in treatment-resistant outpatients with acute exacerbated schizophrenia

**DOI:** 10.3389/fphar.2023.1166507

**Published:** 2023-04-21

**Authors:** Xiaoe Lang, Xiaocui Zang, Feng Yu, Meihong Xiu

**Affiliations:** ^1^ Department of Psychiatry, First Hospital of Shanxi Medical University, Taiyuan, China; ^2^ Qingdao Mental Health Center, Qingdao, China; ^3^ Peking University HuiLongGuan Clinical Medical School, Beijing HuiLongGuan Hospital, Beijing, China

**Keywords:** treatment-resistant, olanzapine, sertraline, negative symptoms, depressive, efficacy

## Abstract

**Background:** Treatment-resistant schizophrenia (TRS) is a major clinical challenge. Current antipsychotic medications do not adequately address negative and depressive symptoms in patients with TRS, and novel treatments are thus needed. This study examines the efficacy of low-dose combined olanzapine (OLA) and sertraline on depressive and negative symptoms in patients with TRS.

**Methods:** A total of 34 TRS outpatients with acutely exacerbated schizophrenia were randomly assigned to OLA monotherapy (12.5–20 mg/day) (control group) or low-dose combined OLA (7.5–10 mg/day) and sertraline (50–100 mg/day) (OS group). Clinical symptoms were assessed using the Positive and Negative Syndrome Scale (PANSS) at baseline and at the end of treatment in weeks 4, 8, 12, and 24. Depressive symptoms and social functioning were also assessed.

**Results:** Compared to the control group, the OS group showed significant improvements in depressive and negative symptoms over time. In addition, the low-dose combination of OLA and sertraline significantly improved social functioning compared with OLA monotherapy. There were no significant between-group differences in psychotic symptom improvement. However, the reduction in Hamilton Depression Rating Scale total score and PANSS negative subscore were not associated with improvements in social functioning, suggesting that these effects of combined treatment are independent.

**Conclusion:** Low-dose combined OLA and sertraline may be effective in the treatment of negative and depressive symptoms compared with standard OLA monotherapy in patients with TRS who are experiencing an acute exacerbation of schizophrenia.

**Clinical Trial Registration**: [ClinicalTrials.gov], identifier [NCT04076371].

## 1 Introduction

To date, the efficacy of antipsychotic medications for the clinical treatment of schizophrenia has been unsatisfactory. Approximately 20–30% of patients with schizophrenia who respond poorly to current antipsychotics are considered to have treatment-resistant schizophrenia (TRS) (Lehman et al., 2004; Howes et al., 2017). For these patients, treatment remains a major challenge. Kane et al. and others have shown that clozapine is superior to other antipsychotics in TRS (Kane et al., 1988; Baig et al., 2021; Correll and Howes, 2021; Zhu et al., 2022). However, its use in clinical practice is limited by barriers including non-response in some patients, severe metabolic side effects, and the need for weekly monitoring of plasma leukocytes and granulocytes for the first 6 months of treatment (Henderson, 2001; Wirshing et al., 2002; Molden, 2021). These effects have led to increased non-psychiatric morbidity and mortality in patients with TRS who are on long-term clozapine treatment by clinical management.

In particular, a recent meta-analysis reported that clozapine is effective in schizophrenia in general, regardless of treatment resistance (Mizuno et al., 2020). However, the limitations of clozapine call into question its exclusive therapeutic role in refractory schizophrenia. The cumulative data suggest that exploring the efficacy of other short- and long-term TRS therapies is warranted. While no other antipsychotic drug has been consistently found to be as effective as clozapine for TRS, a few studies have compared the clinical efficacy of olanzapine (OLA) for this purpose. Volavka et al. (2002) found that, in patients with a history of suboptimal treatment response, OLA and clozapine had modest effects on symptoms of psychosis compared with haloperidol. In their 6-week, double-blind clinical trial of 526 patients with TRS, Breier and Hamilton reported that OLA showed superior efficacy compared with haloperidol (Breier and Hamilton, 1999).

Negative symptoms are typically associated with limited medication response and account for the largest proportion of long-term disability and adverse patient outcomes (Milev et al., 2005; Galderisi et al., 2018; Correll and Schooler, 2020). The association between negative symptoms and significant deficits in motivation, emotion, and social functioning suggests that functional outcomes are significantly affected in schizophrenia (Foussias and Remington, 2010). Negative and depressive symptoms have also been linked (Müller et al., 2006). Indeed, depression is a common comorbidity during the disease course of schizophrenia, affecting approximately 50% of first-episode patients, although with a marked variance in depressive symptoms (Dai et al., 2018; Herniman et al., 2019). Depressive symptoms also influence daily activities and social functioning, which are usually associated with poorer employment status, poorer quality of life, greater suicide risk, and increased relapse risk (Abramowitz et al., 2014; Akinsulore et al., 2014). In patients with schizophrenia, depressive symptoms are associated with worse outcomes (Cohen and Ryu, 2015). Thus, negative and depressive symptoms in schizophrenia represent an unmet therapeutic need.

Several clinical trials have demonstrated that a combination treatment strategy using an antipsychotic and a so-called “‘add-on’” antidepressant can improve negative and depressive symptoms without severe side effects in patients with these persistent symptoms who are resistant to antipsychotic monotherapy (Silver and Nassar, 1992; Spina et al., 1994; Goff et al., 1995; Jockers-Scherübl et al., 2005). A meta-analysis found significant differences in negative symptoms with antidepressant and antipsychotic combination treatment, compared with antipsychotic monotherapy, in patients with schizophrenia (Rummel et al., 2005). However, combination treatment means higher total doses, potential drug interactions, higher costs, and a higher likelihood of treatment non-adherence. Sertraline is a widely used selective serotonin reuptake inhibitor (SSRI) that increases extracellular dopamine concentrations in the nucleus accumbens and striatum (Kitaichi et al., 2010). For these reasons, we sought to assess low-dose combined OLA and sertraline as novel clinical treatment strategies to reduce negative and depressive symptoms with comparable efficacy to standard doses for psychotic symptoms.

This 24-week randomized controlled clinical trial examined the efficacy of low-dose combined OLA and sertraline in patients with TRS. We hypothesized that there would be significant improvements in negative and depressive symptoms in TRS patients with acute exacerbation schizophrenia after combined treatment with OLA and sertraline compared to OLA monotherapy.

## 2 Methods

### 2.1 Patients

From January 2016 to June 2017, 50 outpatients from First Hospital of Shanxi Medical University in China were screened. Eligible adult patients (18–60 years old) with schizophrenia who had experienced acute symptom exacerbation within the past 14 days were recruited. Of these, 16 were excluded, and the remaining 34 patients were randomized to two treatment groups. The reasons for exclusion included not meeting inclusion criteria (*n* = 10), refusal to participate (*n* = 2), and other reasons (*n* = 4). Patients or their guardians provided written informed consent at the start of this clinical trial. The protocol of this study was reviewed and approved by the Ethics Committee of First Hospital of Shanxi Medical University.

**FIGURE 1:**
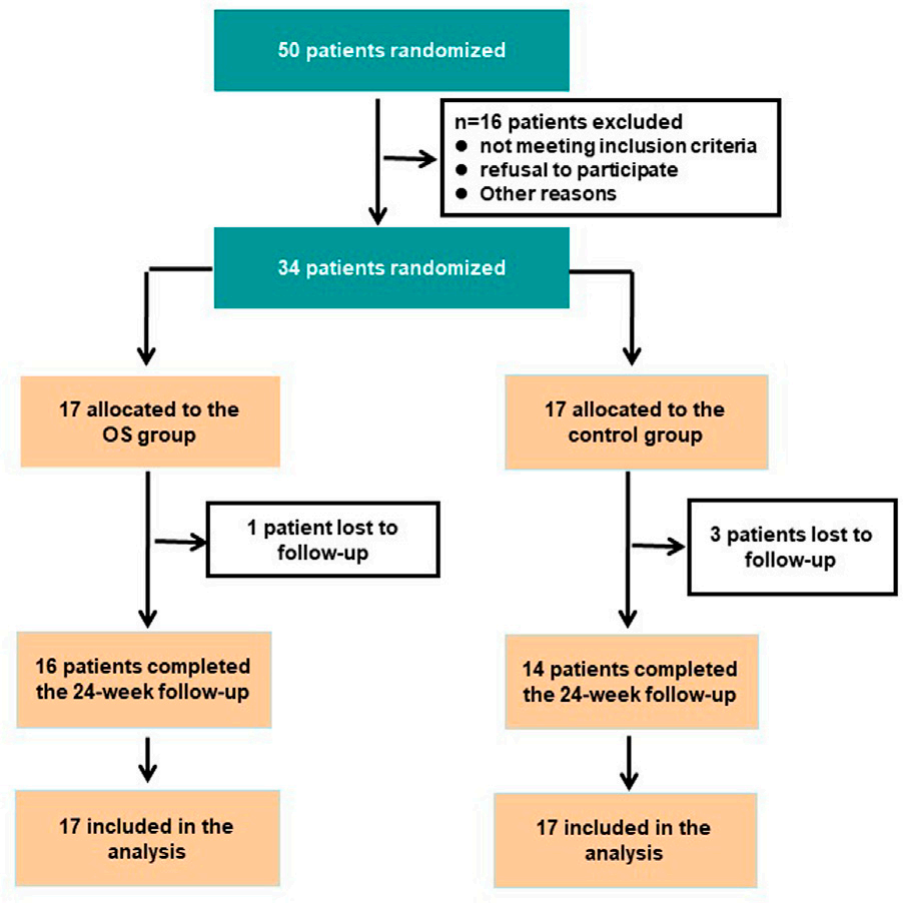
CONSORT flow diagram.

The diagnosis was established by an experienced clinician according to the Diagnostic and Statistical Manual of Mental Disorders, Fourth Edition (DSM-IV) and the Structured Clinical Interview for DSM Disorders (SCID). Treatment resistance was determined according to the criteria of Howes et al. (2017): continued psychosis despite at least two treatment courses with typical antipsychotics at a dose equivalent of chlorpromazine ≥800 mg/day for 6 weeks and post-treatment failure to reduce the Brief Psychiatric Rating Scale (BPRS) total score by ≥ 20%, a BPRS score of ≤45, or a Clinical Global Impressions-Severity (CGI-S) score by > 4. The other inclusion criterion was a lack of stable mental or social functioning during the past 5 years. The key exclusion criteria were: 1) active substance use disorder (excluding tobacco); 2) any clinically significant somatic illness (e.g., diabetes, hypertension, thyroid dysfunction, and brain tumor) potentially related to psychiatric symptoms; 3) self-injury, destructive behavior, or suicidal behavior; 4) a diagnosis of any mental disorder other than schizophrenia; 5) pregnancy or plans to become pregnant within the next 24 weeks; and6) abnormal routine biochemical parameters at admission. A total of 34 patients with TRS who were experiencing acute psychosis exacerbation were recruited (Figure 1).

### 2.2 Intervention

This study is part of a larger one that aims to recruit a total of 1,640 patients with schizophrenia to compare the efficacy of OLA *versus* OLA plus sertraline and similar comparisons for other antipsychotics, including risperidone, paliperidone, and ziprasidone. For this 24-week, single-randomized controlled trial, the combination treatment group included 17 patients who received oral OLA (7.5–10 mg/day) plus sertraline (50–100 mg/day) tablets twice daily (the OS group), and the control group included 17 patients who received oral OLA (12.5–20 mg/day) monotherapy tablets twice daily. The OLA and sertraline dosages in both groups were flexible, according to the psychiatrist’s optimization. Patients on other antipsychotic medications were allowed 1 week of transition overlap prior to study entry. Patients in both groups were allowed to take 2–3 mg/d oral clonazepam during the first 4 weeks of treatment to improve sleep and reduce anxiety and acute agitation. No other psychotropic medications (anticholinergics and mood stabilizers) were used in this study.

### 2.3 Blinding and randomization

Patients were randomly assigned using a 1:1 allocation ratio to the OS or control group. Computer-generated random sequences in blocks of six patients were stored in opaque, sealed envelopes until a nurse who had no contact with the assessors, or any other study involvement used these to assign each participant. Neither assessors nor patients were informed of the random number or assignment.

### 2.4 Psychiatric symptoms assessments

Depressive symptoms were assessed by the Hamilton Depression Rating Scale (HAMD) (Hamilton, 1960). Other outcome measures included the Positive and Negative Syndrome Scale (PANSS) (Kay et al., 1987), the CGI-S (Haro et al., 2003), and the Personal and Social Performance Scale (PSP) (Morosini et al., 2000). Three experienced assessors were trained prior to the study. After training, the interobserver correlation coefficients for the PANSS total score and PSP score were maintained at >0.8. Each patient was assessed by a single rater throughout the trial.

### 2.5 Statistical analysis

Demographic and clinical characteristics at baseline were analyzed via analysis of variance (ANOVA) or the Χ^2^ test. Data missing for patients who dropped out were imputed via the last observation carried forward (LOCF) method. Repeated-measures ANOVA was used as the primary analysis, with an intention-to-treat (ITT) design used to evaluate between-group differences. In the model, outcomes (five-time points) were entered as the within-effect, and treatment groups (two levels) were used as the between-effect. We focused on the interaction between the treatment group and follow-up time points. When significant, *post hoc* tests with Bonferroni correction were used to compare the baseline and follow-up outcomes via analysis of covariance (ANCOVA). The ANCOVA was also used to compare outcomes at follow-up time points with the baseline score as between-group covariates. Finally, regression analysis was conducted to identify factors predictive of improvements in social functioning in the OS group. The independent model factors were changes in HAMD and PSP total scores from baseline to week 24. Independent variables included age, gender, illness duration, and change in PANSS total score.

Bonferroni corrections were performed to adjust for multiple comparisons. The threshold for statistical significance was *p* < 0.05.

## 3 Results

### 3.1 Descriptive statistics

Participants’ gender, age, and education were typical of patients with TRS. Thirty-four outpatients were randomized to one of the two study groups (17 to each). Four patients were lost to follow-up due to significant side effects (one in the OS group and three in the control group) (Figure 1). No patients who consented to the study protocol dropped out prior to randomization due to the combination therapy strategy.

We found no significant group differences in age, years of education, age of illness onset, or gender (all *p* > 0.05). Those in the OS group had higher score in PANSS total scores compared with those in the control group (*p* = 0.04) (Table 1).

**TABLE 1:** Demographic and clinical characteristics in the combination therapy group (OS) and control group (mean ± SD).

Variable	OS group (*n* = 17)	Control group (*n* = 17)	F or *X* ^2^ (*p*-value)
Demographic data			
Gender (male patients/female patients)	6/11	9/8	1.4 (0.24)
Age (years)	43.9 ± 8.2	47.2 ± 6.5	1.6 (0.21)
Education (years)	10.5 ± 2.0	10.7 ± 2.0	0.06 (0.80)
Clinical data			
Duration of illness (years)	12.6 ± 4.9	13.9 ± 5.8	0.5 (0.50)
Age of onset (years)	30.2 ± 5.6	32.1 ± 4.9	1.1 (0.30)
P subscore	22.6 ± 4.6	26.2 ± 8.5	2.3 (0.14)
N subscore	29.4 ± 6.6	30.9 ± 10.5	0.3 (0.62)
G subscore	58.6 ± 8.1	63.6 ± 11.6	2.1 (0.16)
PANSS total score	110.7 ± 11.7	120.6 ± 14.8	4.7 (0.04)
HAMD total score	22.5 ± 4.7	26.0 ± 5.6	3.7 (0.06)
PSP total score	30.6 ± 5.0	31.0 ± 5.9	0.02 (0.90)

Note: *SD*, standard deviation; *P*, positive subscore; *N*, negative subscore; *G*, general psychopathology subscore; *CGI-S*, Clinical Global Impression-Severity; *PSP*, Personal and Social Performance Scale; *HAMD*, Hamilton Depression Rating Scale.

### 3.2 Effects on psychotic symptoms

**FIGURE 2:**
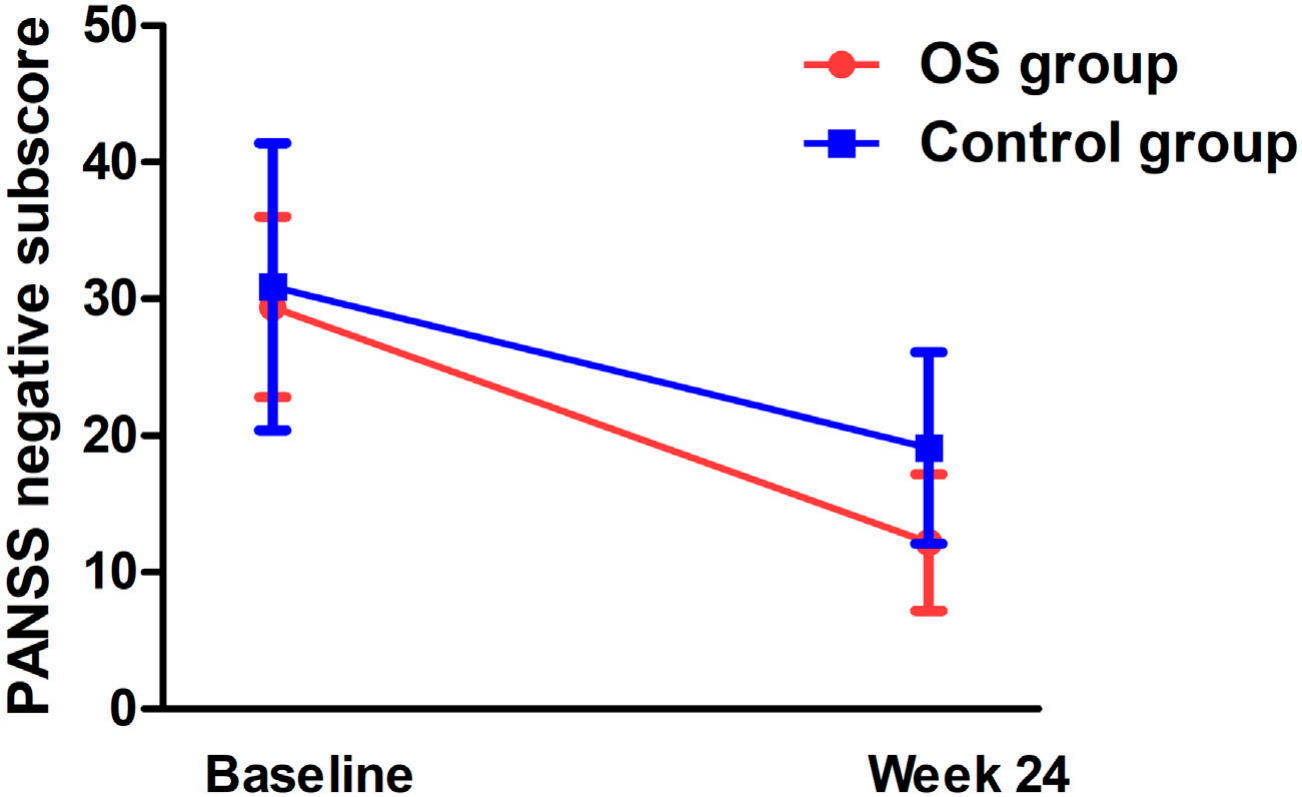
Interaction effect of group and time on negative symptoms in TRS patients (*p* < 0.05).

Significant group-by-time effects were observed on the negative subscore (F = 7.9, p_Bonferroni_ = 0.016) (Figure 2) and general subscore (F = 7.5, p_Bonferroni_ = 0.012) (Table 2). After controlling for baseline ratings for the PANSS total score, significant differences were observed in negative symptoms and general psychopharmacology between the OS and control groups from weeks 4 to 24 (Table 2). Significant differences were also observed in the OS group between baseline and follow-up on negative symptoms and general psychopharmacology starting at week 4 and continuing throughout the study period (Table 3).

**TABLE 2:** PANSS scores, HAMD score, CGI-S, and PSP at baseline, weeks 4, 8, 12, and 24 in the patients treated with olanzapine plus sertraline (OS) group and olanzapine monotherapy (control) group (mean ± standard deviations).

	Week 4 (*n* = 34)	Week 8 (*n* = 34)	Week 12 (*n* = 34)	Week 24 (*n* = 34)	Group F (*p*-value)	Group×Time F (*p*-value)
***Positive subscore***					3.8 (0.06)	3.4 (0.08)
OS group	17.5 ± 3.6	12.2 ± 3.3	9.8 ± 2.7	9.1 ± 2.5		
Control group	18.6 ± 6.7	14.8 ± 6.1	11.5 ± 4.3	10.6 ± 4.2		
***Negative subscore***					2.6 (0.12)	10.6 (0.003)
OS group	24.5 ± 6.8	19.3 ± 6.6	16.5 ± 6.2	12.2 ± 5.0		
Control group	28.2 ± 10.8	25.5 ± 10.0	22.1 ± 8.8	19.1 ± 7.9		
***General psychological subscore***					4.1 (0.052)	1.9 (0.18)
OS group	48.1 ± 8.2	38.1 ± 8.6	25.5 ± 7.4	23.1 ± 8.0		
Control group	55.9 ± 8.4	44.7 ± 8.6	27.7 ± 6.7	24.5 ± 7.6		
***PANSS total score***					10.2 (0.003)	0.5 (0.48)
OS group	89.2 ± 13.1	69.6 ± 14.4	51.6 ± 14.1	44.1 ± 14.8		
Control group	101.2 ± 13.4	83.8 ± 12.7	61.0 ± 11.4	53.3 ± 13.8		
***CGI-S score***					11.6 (0.002)	10.5 (<0.001)
OS group	4.8 ± 0.4	3.9 ± 0.4	3.0 ± 0.7	2.5 ± 0.8		
Control group	5.1 ± 0.5	4.2 ± 0.6	3.5 ± 0.9	3.6 ± 0.6		
***HAMD total score***					40.1 (<0.001)	8.1 (0.008)
OS group	16.2 ± 4.2	10.6 ± 4.2	8.0 ± 4.6	5.8 ± 5.3		
Control group	22.9 ± 4.6	18.5 ± 4.5	14.8 ± 3.6	13.5 ± 3.6		
***PSP total score***					7.6 (0.01)	27.5 (<0.001)
OS group	43.1 ± 6.5	60.8 ± 10.8	74.4 ± 13.7	84.4 ± 3.8		
Control group	41.4 ± 6.5	56.8 ± 9.8	62.8 ± 10.6	68.5 ± 12.2		

Note: controls for age, gender, onset age, and disease course.

**TABLE 3:** Psychiatric symptoms and psychosocial functioning at baseline and weeks 4, 8, 12, and 24 in the combination treatment and monotherapy groups.

Parameter	Change from baseline to week 4		Change from baseline to week 8		Change from baseline to week 12		Change from baseline to week 24	
	OS	Control	OS	Control	OS	Control	OS	Control
*P subscore*								
Mean	5.6	8.3	11.1	12.3	13.8	16.1	14.9	17.7
SD	2.1	2.4	1.8	3.1	2.9	5.5	3.6	7.1
*N subscore*								
Mean	4.9	2.8	10.1	5.5	12.9	8.8	17.2	11.9
SD	1.6	0.9	2.9	1.9	4.1	3.3	6.8	5.1
*G subscore*								
Mean	10.6	7.7	20.6	18.9	33.2	35.9	35.6	39.1
SD	3.9	9.3	6.0	10.6	10.5	14.9	11.2	15.9
*PANSS total score*								
Mean	21.5	19.4	41.1	36.8	59.1	59.6	66.6	67.3
SD	6.5	6.9	11.1	12.0	16.5	19.6	19.4	23.2
*CGI total score*								
Mean	0.94	0.4	1.9	1.4	2.9	1.6	3.5	2.1
SD	0.7	0.5	0.7	0.8	0.7	1.0	0.5	0.7
*HAMD total score*								
Mean	6.4	3.1	11.9	7.5	14.5	11.2	16.8	12.5
SD	2.5	3.2	4.4	4.0	5.4	4.6	5.8	5.6
*PSP total score*								
Mean	12.4	10.4	30.2	25.8	43.8	31.8	53.8	37.5
SD	5.0	4.9	9.5	9.5	12.0	9.7	14.4	11.2

Note: *SD*, standard deviation; *P*, positive subscore; *N*, negative subscore; *G*, general psychopathology subscore; *CGI-S*, Clinical Global Impression-severity; *PSP*, Personal and Social Performance Scale; *HAMD*, Hamilton Depression Rating Scale.

### 3.3 Effects on depressive symptoms

**FIGURE 3:**
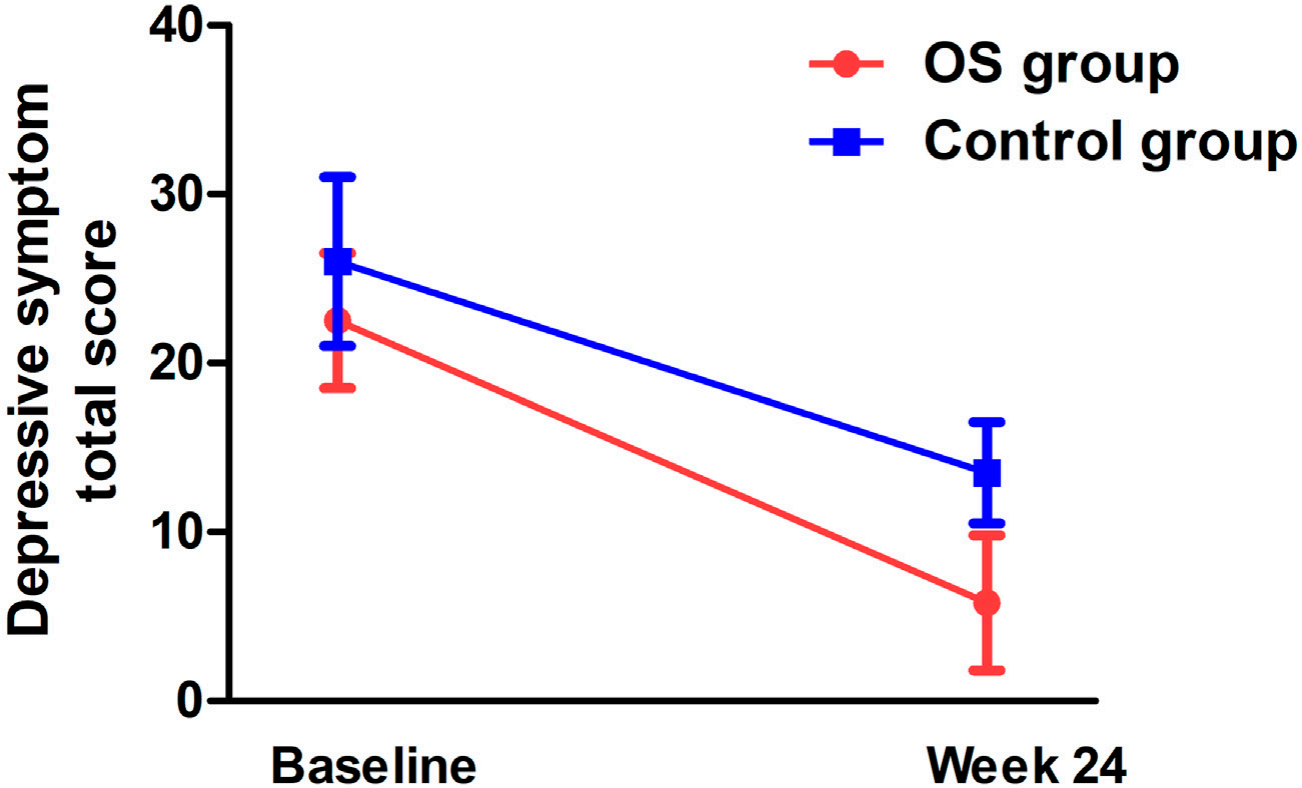
Interaction effect of group and time on depressive symptoms in TRS patients (*p* < 0.05).

There was a significant interaction effect between time and treatment group on the HAMD total score (F = 5.2, p_Bonferroni_= 0.044) (Table 2; Figure 3). After controlling for age, education, and gender, the interaction effect remained statistically significant (p_Bonferroni_= 0.05). Further analysis showed that the differences from follow-up weeks 4 through 24 were significant in the OS group. After controlling for baseline ratings for the HAMD total score, significant differences in depressive symptoms were observed between the OS and control groups from weeks 4 to 24.

### 3.4 Effects on social functioning

Repeated measures ANOVA showed an interaction effect between group and time (F = 31.1, p_Bonferroni_= 0.001) on the PSP score (Table 2). The combination treatment improved social functioning significantly compared with the control group. A significant PSP score difference was observed in the fourth follow-up week in the OS group. There were also significant between-group differences from week 4 onward, after controlling for baseline ratings for the PSP score.

### 3.5 Associations between changes in psychotic symptoms and social functioning

The OS group showed a significant association between PANSS total score and PSP score at baseline (r = 0.73, *p* = 0.001). However, there was no significant association between PANSS total score improvement and either the PSP total score or the HAMD total score at any of weeks 4, 8, 12, or 24 (all *p* > 0.05).

## 4 Discussion

We found that: 1) low-dose combined OLA and sertraline was more effective in treating negative symptoms than OLA alone; 2) combination treatment significantly improved depressive symptoms better than OLA alone; and 3) patients in the combination treatment group showed significant improvements in social functioning compared with the control group.

To the best of our knowledge, this is the first study to show that low-dose combined OLA and sertraline are effective in reducing negative symptoms in patients with TRS. There is clear evidence that patients with TRS have persistent, long-term, moderate-to-severe negative symptoms (Kane and Correll, 2016), which negatively impact their health-related quality of life. Although OLA is more effective than first-generation antipsychotics in ameliorating negative symptoms in patients with schizophrenia, OLA alone is not particularly effective for negative symptoms (Leucht et al., 2009). Our findings are consistent with previous studies suggesting that adjunctive antidepressants are beneficial in alleviating negative symptoms in patients with TRS who are experiencing acute symptom exacerbation while on stable antipsychotic treatment (Cho et al., 2011; Galling et al., 2018).

We speculate that the pharmacological mechanism of sertraline’s negative symptom improvements may be related to its modulation of the serotonin (5-HT) system and dopamine signaling pathway in patients with TRS. Evidence suggests the pathological mechanisms of TRS, including the abnormal functioning of dopaminergic, 5-HT, and other neurotransmitter pathways (Demjaha et al., 2014). In particular, negative symptoms have been linked to relative dopamine deficiency and 5-HT insufficiency in the prefrontal cortex. Sertraline, which has been used here and is a widely used SSRI, plays a role in both the 5-HT and norepinephrine neuronal systems (MacQueen et al., 2001; Cleare et al., 2015). However, sertraline has more dopaminergic activity than other SSRIs (Singh and Saadabadi, 2022).

Consistent with our expectations, depressive symptoms improved significantly with low-dose combined OLA and sertraline in patients with TRS. This finding is consistent with another report that showed that combination treatment with OLA plus sertraline for 12 weeks was effective in treating depressive symptoms in depressed patients with psychotic symptoms (Meyers et al., 2009), although there is a notable difference between these patient samples (i.e., schizophrenia vs. depression). Sertraline as an adjunct to antipsychotic medication has been reported to alleviate depressive symptoms in patients with schizophrenia (Gregory et al., 2017), although this is rarely recommended in clinical practice guidelines due to a lack of high-quality efficacy evidence (Buchanan et al., 2010; Zink et al., 2010; Galling et al., 2018).

Few studies have found that antidepressant and antipsychotic combination treatment is an option for patients with schizophrenia, including the combination of OLA and sertraline. Our study provides novel evidence that low-dose combined OLA and sertraline may be effective in treating depressive symptoms in patients with TRS. It should be noted that the doses of sertraline and OLA used here were relatively low. Sertraline 50 mg/day accounts for approximately 80% of the 5-HT transporter, and further dose increases do not provide significant efficacy (Meyer et al., 2004; Furukawa et al., 2020); this may be why dose reductions did not affect efficacy. It should also be noted that negative and depressive symptoms may also overlap in symptomatology, neurocognition, neurobiology, and genetics, making them difficult to distinguish clinically (Siris, 2000).

The low-dose combination of OLA and sertraline also significantly improved social functioning. Social functioning, an essential and increasingly important outcome measure in schizophrenia, can be significantly reduced by the presence of negative and depressive symptoms (Vita and Barlati, 2018). However, few previous studies have assessed this outcome. Thus, our finding of improvement in social functioning following low-dose combined treatment with OLA and sertraline may have important clinical implications. Although we found a significant association between clinical symptom severity and social functioning at baseline, we did not find an association between clinical symptom improvement and social functioning. Therefore, the combination treatment may have improved social functioning independently of clinical improvement.

Finally, low-dose combination treatment had comparable efficacy on psychotic symptoms to OLA monotherapy in patients with TRS. This finding is inconsistent with previous clinical trials that evaluated inpatients with acute psychosis; these showed that antipsychotic and antidepressant combination treatment delayed the reduction in hallucinations (Kramer et al., 1989) and exacerbated symptoms of psychosis (Lehman et al., 2004). This inconsistency may be due to differences in clinical characteristics (e.g., medication history). In contrast, our study is consistent with a recent meta-analysis of psychosis exacerbation in a relatively large sample (N = 725), which reported a risk ratio close to 1 (1.03, 95% confidence interval = 0.60–1.75), suggesting no adverse effects of combined antidepressants on psychotic symptoms (Helfer et al., 2016). This meta-analysis also reported that positive symptoms were slightly improved rather than worsened by antidepressants—likely a secondary effect of mood improvement. Another review analyzed the efficacy of adjunctive antidepressants to treat psychiatric symptoms and side effects from 36 randomized, controlled trials and found that this approach did not worsen the course of psychosis (Terevnikov et al., 2015).

It should be noted that the two treatment groups had significantly different baseline ratings for PANSS total scores, although negative and depressive symptoms were not significantly different. Patients in the control group had higher PANSS total scores than those in the OS group, indicating that the control group presented with more severe psychotic symptoms at enrollment. Although we have tried to minimize the effects of different baseline ratings by using the baseline ratings for PANSS total scores as covariates, this baseline difference may bias our findings. In addition, due to the relatively small sample with TRS and acute exacerbation schizophrenia here, further studies with large, matched demographics and baseline symptom severity are warranted to verify these findings.

Our randomized clinical trial had several limitations. First, its statistical power was limited by a relatively small sample, which may have contributed to bias. Further clinical trials with larger samples are therefore needed. Second, this clinical trial was not double-blinded, which limits the validity of this study. Although the interviewers were blinded, there was no blinding for the addition of sertraline. Patients in the OS group took two capsules, while those in the control group took one. Thus, participants may have been able to recognize their group assignment through personal communication. Third, 24 weeks is a relatively short-term follow-up, which may have prevented a full investigation of combination treatment efficacy for negative and depressive symptoms. Fourth, we did not compare the efficacy of low-dose combined OLA and sertraline with clozapine, which is commonly prescribed for TRS. Fifth, patients’ negative symptoms were not assessed with a specific scale (e.g., the Brief Negative Symptom Scale (BNSS) or the Clinical Assessment Interview for Negative Symptoms (CAINS)) and depressive symptoms were not assessed with the Calgary Depression Scale for Schizophrenia (CDSS ), the gold standard for assessing depressive symptoms in schizophrenia.

## 5 Conclusions

Low-dose OLA plus sertraline is more effective for negative and depressive symptoms than OLA monotherapy in patients with TRS who are experiencing an acute exacerbation of symptoms. This study provides evidence for a new treatment option for this patient population that may alleviate negative and depressive symptoms without impacting the efficacy of psychotic symptoms. To the best of our knowledge, this study is the first to report the therapeutic efficacy of low-dose combined OLA and sertraline on negative and depressive symptoms simultaneously. Despite these encouraging findings, replication with larger samples is needed.

## Data Availability

The raw data supporting the conclusion of this article will be made available by the authors, without undue reservation.
